# Systematic Analysis of Monoallelic Gene Expression and Chromatin Accessibility Across Multiple Tissues in Hybrid Mice

**DOI:** 10.3389/fcell.2021.717555

**Published:** 2021-09-23

**Authors:** Weizheng Liang, Xudong Zou, Guipeng Li, Shaojie Zhou, Chi Tian, Bernhard Schaefke

**Affiliations:** ^1^Harbin Institute of Technology, Harbin, China; ^2^Shenzhen Key Laboratory of Gene Regulation and Systems Biology, School of Life Sciences, Southern University of Science and Technology, Shenzhen, China; ^3^Department of Biology, Southern University of Science and Technology, Shenzhen, China; ^4^Academy for Advanced Interdisciplinary Studies, Southern University of Science and Technology, Shenzhen, China

**Keywords:** monoallelic, allelic, gene expression, chromatin accessibility, *cis* regulation, hybrid mice

## Abstract

In diploid eukaryotic organisms, both alleles of each autosomal gene are usually assumed to be simultaneously expressed at similar levels. However, some genes can be expressed preferentially or strictly from a single allele, a process known as monoallelic expression. Classic monoallelic expression of X-chromosome-linked genes, olfactory receptor genes and developmentally imprinted genes is the result of epigenetic modifications. Genetic-origin-dependent monoallelic expression, however, is caused by *cis*-regulatory differences between the alleles. There is a paucity of systematic study to investigate these phenomena across multiple tissues, and the mechanisms underlying such monoallelic expression are not yet fully understood. Here we provide a detailed portrait of monoallelic gene expression across multiple tissues/cell lines in a hybrid mouse cross between the *Mus musculus* strain C57BL/6J and the *Mus spretus* strain SPRET/EiJ. We observed pervasive tissue-dependent allele-specific gene expression: in total, 1,839 genes exhibited monoallelic expression in at least one tissue, and 410 genes in at least two tissues. Among these 88 are monoallelic genes with different active alleles between tissues, probably representing genetic-origin-dependent monoallelic expression. We also identified six autosomal monoallelic genes with the active allele being identical in all eight tissues, which are likely novel candidates of imprinted genes. To depict the underlying regulatory mechanisms at the chromatin layer, we performed ATAC-seq in two different cell lines derived from the F1 mouse. Consistent with the global expression pattern, cell-type dependent monoallelic peaks were found, and a higher proportion of C57BL/6J-active peaks were observed in both cell types, implying possible species-specific regulation. Finally, only a small part of monoallelic gene expression could be explained by allelic differences in chromatin organization in promoter regions, suggesting that other distal elements may play important roles in shaping the patterns of allelic gene expression across tissues.

## Introduction

Protein-coding information stored in DNA is first transcribed to mRNA and then translated into polypeptide chains. Knowing how these processes are regulated is critical for the understanding of development and evolution. Indeed, divergence in gene expression is considered a major cause of phenotypic differences between species (King and Wilson, 1975). Transcriptional regulation is mediated by the interaction between *cis*-regulatory elements (e.g., promoters and enhancers) and *trans*-factors (e.g., transcription factors (TFs)). Whereas *cis*-elements are usually located within or nearby a single target gene whose gene expression they regulate, *trans*-factors can be located on different chromosomes and potentially influence the expression of several often distal target genes. Besides quantitative trait loci (QTL) mapping analyses, which require large sample sizes and inform about distal and proximal elements affecting gene expression differences, F1 hybrid studies are another widely applied and more straightforward approach to distinguish between *cis* and *trans* acting regulatory components (Wittkopp et al., 2004; Gao et al., 2015; Hou et al., 2015; Xiao et al., 2016; Xu et al., 2017). With two alleles sharing the same *trans* environment, allelic differences in the F1 hybrid can be directly interpreted as *cis*-regulatory divergence (Gao et al., 2015; Hou et al., 2015; Xiao et al., 2016). By comparing these allelic-specific variations with the differences between parental strains or species, the *trans*-component of gene expression differences can be estimated (Wittkopp et al., 2004, 2008; Goncalves et al., 2012; Wong et al., 2017). The F1 hybrid approach has been used to study *cis* and *trans* regulatory divergence contributing to differences in gene expression between strains of the same species or closely related species in many model organisms, including yeast (Tirosh et al., 2009; Emerson et al., 2010; Schaefke et al., 2013), Drosophila (Wittkopp et al., 2004) and mouse (Goncalves et al., 2012). F1 hybrid studies of different *Mus musculus* subspecies revealed pervasive *cis*-regulatory differences but comparatively few *trans*-regulatory differences (Goncalves et al., 2012; Crowley et al., 2015). However, the interplay of these two kinds of elements shaping the regulatory patterns of gene expression divergence across tissues in mammals has not been fully understood.

The most extreme case of an allelic-biased expression pattern is monoallelic expression (when a gene is only transcribed from one of the two parental alleles). Classic monoallelic expression of X-chromosome-linked genes, olfactory receptor genes and developmentally imprinted genes is the result of epigenetic modifications (Chess, 2013, 2016; Eckersley-Maslin et al., 2014; Gendre et al., 2014). Genetic-origin-dependent monoallelic expression, in contrast, is caused by *cis*-regulatory differences between the alleles (Ohishi et al., 2020), and cases of non-random allele-dependent X-chromosome inactivation have also been described (Orstavik et al., 1995; Thorvaldsen et al., 2012; Calaway et al., 2013; Jones, 2014). However, the tissue-dependence of these phenomena is rather underexplored, and the mechanisms underlying asymmetric expression are not yet fully understood.

Here we provide a detailed portrait of monoallelic gene expression across multiple tissues/cell lines in a hybrid mouse model and allelic chromatin accessibility patterns in two different cell lines. We focus on depicting tissue-dependent allele-specific gene expression patterns and the underlying regulatory mechanisms at the chromatin accessibility layer. We observed pervasive tissue-dependent allele-specific gene expression and chromatin accessibility patterns. In total, 1,839 genes exhibited monoallelic expression in at least one tissue, and 410 in at least two tissues. We identified six autosomal monoallelic genes with the active allele being identical in all eight tissues, which are likely novel candidates of imprinted genes. Also, we found 88 monoallelic genes with different active alleles between tissues. Only a small part of monoallelic gene expression could be explained by allelic chromatin structural differences in promoter regions, suggesting that other distal elements or differential TF binding without divergence in chromatin remodeling may play important roles in shaping the patterns of allelic gene expression across tissues.

## Materials and Methods

### RNA-Seq Data of F1 Hybrid Mouse

RNA-seq raw data of F1 hybrid mice containing six organs, embryonic stem cells (ESCs), and fibroblasts were obtained from previous studies (Gao et al., 2015; Zou et al., 2021). For each tissue/cell type, raw sequencing data of two biological replicates were downloaded. Samples from heart, kidney and cortex were sequenced with paired-end reads of 101 bp length. Samples from spleen, lung and ESCs were sequenced with paired-end reads of 76 bp length. The sequencing depth for each biological replicate was 240–260 million reads per sample, except for the two ESC samples, for which we obtained 175 million and 202 million reads, respectively.

### Assay for Transposase-Accessible Chromatin-Seq Library Construction and Sequencing

The ATAC-seq libraries of F1-ESCs and F1-fibroblasts, each with three biological replicates, were prepared as previously described with minor modifications (Corces et al., 2017; Liang et al., 2021). Briefly, 50,000 fresh cells were lysed in lysis buffer for 10 min on ice to prepare the nuclei. Immediately after lysis, nuclei were spun at 500 g for 10 min to remove the supernatant. Nuclei were then incubated with the Tn5 transposase (Vazyme) in tagmentation buffer at 37°C for 30 min. After tagmentation, PCR was performed to amplify the library for 12 cycles under the following PCR conditions: 72°C for 3 min; 98°C for 30 s; and thermocycling at 98°C for 15 s, 60°C for 30 s, and 72°C for 40 s; followed by 5 min at 72°C. After the PCR reaction, libraries were purified with DNA purification beads (Vazyme). The libraries were sent to Annoroad for sequencing and 2 × 150 bp reads were obtained.

### RNA-Seq Data Processing

The reference *M. musculus* genome (mm10) and gene annotation of the C57BL/6J strain were downloaded from the Ensemble database^1^ (version: GRCm38, release 74). SNVs and insertions/deletions (indels) between C57BL/6J and SPRET/EiJ were downloaded from the Mouse Genome Project.^2^

The vcf2diploid tool (version 0.2.6) in the AlleleSeq pipeline (Rozowsky et al., 2011) was used to construct the SPRET/EiJ genome by incorporating the SNVs and indels into the C57BL/6J genome. The chain file between the two genomes was also reported as an output, which was further used with g2gtools to convert SPRET/EiJ coordinates to C57BL/6J coordinates.

To ensure that RNA-seq reads from all tissues have the same length, we trimmed 25 bp from the 3′ end of the 101 bp reads. We aligned the RNA-seq reads to the C57BL/6J reference genome and SPRET/EiJ genome separately with HISAT2 (version 2.0.1) with parameters -p 12 -k 2 –reorder –no-softclip (“softclip” was not allowed when mapping in order to avoid junction reads to be cut off; the “reorder” parameter was used to ensure that the reads order of the mate pairs in the HISAT2 output is consistent with the order of reads of the input file for efficient assignment). Reads were assigned to the genome with less mapping edit distance. The reads with equal mapping distance to both genomes were assigned as common reads. Genomic alignment coordinates of the reads that were assigned to SPRET/EIJ were then converted to the corresponding locations in the C57BL/6J reference genome using the g2gtools software (version 0.1.29). The bias of allelic reads assignment in favor of the reference genome (C57BL/6J) was low, ranging from 0.1% in kidney to 3.5% in fibroblasts (S), which indicates that our strategy for allelic reads assignment is reliable for allelic gene expression estimation.

### Gene Expression Level Quantification

After reads alignment and allelic reads assignment, uniquely mapped reads of each allele were chosen and fed into featureCounts (v1.6.0) for gene expression quantification. Only both ends of a read pair concordantly mapped were counted (by “-B” and “-C”). Raw read counts were then normalized as transcripts per kilobase million (TPM).

### Identifying Allelic Differential Genes

Divergent and monoallelic genes were detected following the pipeline in Supplementary Figure 1. At first, protein-coding genes with TPM no greater than 1 (not allelic) in both biological replicates, and genes located in X, Y, and mitochondrial chromosomes, as well as known imprinting genes were removed. The remaining autosome protein-coding genes were kept for divergent and monoallelic genes analysis. To make sure the differential analysis between alleles are supported by enough SNVs, we selected genes with 5 or more allele informative SNVs (covered by more than 20 reads) between alleles in all annotated exons. Paired-sample *t*-test on count of reads cross SNVs in a genes were performed and BH adjust *p*-values were obtained. The log2 transformed fold change were calculated between alleles based on summed up reads cross all SNVs. A gene was defined as allelic differential gene (ADE) if LFC greater than 1 and adjusted *p*-value less than 0.05.

### Assay for Transposase-Accessible Chromatin-Seq Data Processing

2 × 150 bp paired-end reads were first trimmed to remove adapter sequences using Trim Galore v0.6.4 (Krueger, 2016) (–cores 4 –paired –nextera –length 50). Cleaned reads were aligned to the C57BL/6J reference genome and SPRET/EiJ genome separately with g Bowtie2 (Langmead and Salzberg, 2012) (version 2.4.1) with parameters -p 8 -X 2000. Reads mapped to the mitochondrial genome and low mapping quality reads (MAPQ < 10) were filtered out using custom scripts. Picard (v2.12.1) was then used to sort the reads and remove duplicates. Reads were assigned to the two mouse genomes with less mapping edit distance. Only reads which could be assigned unambiguously (allelic reads or allele informative reads) to either of the two genomes were kept for further analysis. Genomic alignment coordinates of the reads that were assigned to SPRET/EIJ were then converted to the corresponding locations in the C57BL/6J reference genome using the g2gtools software (version 0.1.29).

Reads of both alleles in each of the six samples were merged as input for MACS2 to call consensus peaks (-f BAMPE -g mm –keep-dup all –nomodel –nolambda -B). And to ensure reproducibility, only peaks detected by IDR (version 2.0.4.2, with parameter –idr-threshold 0.05) in all three replicates were used for further analysis.

After obtaining consensus peaks, allelic read counts for each peak in each sample were analyzed by featureCounts v1.6.4 (Liao et al., 2014) (with parameters: -F SAF -p -B -C -T 4). Differential peaks between alleles were detected using the R package DESeq2 (Love et al., 2014) (under R version 4), and peak annotation was analyzed with the R package ChIPseeker (Yu et al., 2015) (under R version 4).

### Gene Annotation

Gene type, exons, and transcription start site (TSS) annotations were extracted from the gene annotation file of the mouse reference genome mm10 downloaded from the Ensembl website.^3^

### Filtering

Non-coding genes were firstly removed from our gene list, and to ensure reliable downstream analysis, X-chromosomal genes and known imprinted genes^4^ were analyzed separately and only autosomal genes with TPM ≥ 1.0 in both replicates remain. Since allelic reads assignment is dependent on *cis*-variants between alleles, to avoid bias of reads assignment, we further filtered out genes with less than 5 informative SNVs (covered by 20 reads).

### Principal Component Analysis

Principal component analysis (PCA) was performed on all protein-coding genes after filtering. Allelic reads count of each gene in each sample were normalized as counts per million (CPM). Then the normalized count matrix was fed into the R prcomp function to run the PCA analysis, and the first two components were used for sample visualization.

### Allelic Differential Genes and Monoallelic Expression Gene

For each gene, allelic reads covering informative SNVs were summed up, then a logarithm transformed fold change (LFC) between alleles was calculated as in the following Eq. 1:


(1) $$\mathrm{LFC}=\log_{2}\left(\left(\mathrm{BL6}+1\right)/\left(\mathrm{SPR}+1\right)\right),$$

(1) where *BL6* means informative allelic reads from the C57BL/6J allele, and *SPR* means informative allelic reads from SPRET/EiJ allele. Allelic differential genes (ADE) are defined as those with absolute LFC equal or greater than 1, and two-sample paired *t*-test *p*-value < 0.05.

We also defined a *p* score (calculated as the proportion of BL6 allelic reads, Eq. 2) to distinguish monoallelic expression genes (MAE). As defined in a previous study (AV Gendrel, Development cell, 2014), Genes with *p* score >0.85 or *p* score <0.15 were defined as MAEs.


(2) p⁢score=BL6/(BL6+SPR),

where *BL6* means informative allelic reads from the C57BL/6J allele, and *SPR* means reads from the SPRET/EiJ allele.

### Reproducibility of Monoallelic Genes Between Biological Replicates

We used the same cutoff of *p* score (>0.85 or <0.15) as above to define monoallelic patterns in each of the two biological replicates in each tissue. And we calculated the proportion of consistent patterns between the two replicates as shown in Supplementary Table 5. We observed high consistency between replicates for all tissues/cell lines.

### Replication Rate of Monoallelic Genes Between Tissues

To define the replication rate of monoallelic genes between tissues, we calculate a replication rate, similar to the Jaccard Index, which represents the proportion of intersection in the union (Eq. 3). For any two tissues, the replication rate was defined as:


(3) replication⁢rate=⋂MAEs/⋃MAEs,

Where ⋂*MAEs* means monoallelically expressed genes in one tissue, also monoallelically expressed in the other tissue with the same preferred allele; ⋃*MAEs* means the union set of MAE genes between the two tissues.

### *d* Score

We calculated a *d* score for each peak based on the previous definition (Xu et al., 2017). We treated each fibroblast cell line as if derived from a single clone, based on this, peaks of one allele on the X-chromosome would mostly be inactive with a few peaks escaped. Therefore, we compared the distribution of *d* scores in X-inactive peaks and X-escaped peaks, and set the cross-site where the *d* score equals 0.35 as the threshold for defining monoallelic peak.

## Results

### Autosomal Monoallelic Gene Expression Is Pervasive Across Tissues

**FIGURE 1:**
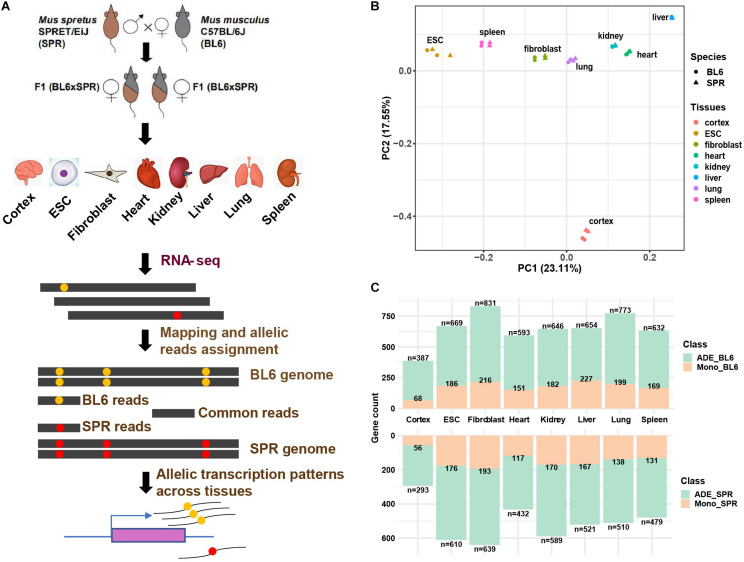
Allelic gene expression profiling across eight tissues/cell types in F1 hybrid mice. **(A)** Scheme of the experimental design and pipeline for allelic gene expression analysis. **(B)** View of the first two principal components of allelic samples. PCA analysis was performed with all expressed genes. Species are indicated with shapes and tissue types are distinguished by colors. **(C)** Allelic differentially expressed (ADE) genes and monoallelic genes in each tissue. The numbers of ADEs and monoallelic genes are indicated in the overhang and within each bar, respectively.

To identify allelic differentially expressed genes in the mouse genome, we performed RNA-seq of six different organs (cerebral cortex, heart, kidney, liver, lung, and spleen) and two cell types (ESCs and fibroblasts) from a highly divergent F1 hybrid cross between the house mouse *M. musculus* (C57BL/6J) and the Algerian mouse *Mus spretus* (SPRET/EiJ) which was generated for previous studies (Gao et al., 2015) in our lab (Figure 1A). Data of two biological replicates of each organ and cell type were used. After read mapping (see section “Materials and Methods”), uniquely mapped read pairs were assigned to each allele based on “edit distance.” PCA analysis shows that the samples are clustered together firstly by tissue type or cell type and then by species (Figure 1B), which is consistent with previous studies (Barbosa-Morais et al., 2012; Merkin et al., 2012). To accurately estimate genes with allelic differential expression (ADE genes) and monoallelic expression (MAE genes), only protein-coding genes in autosomes with at least 5 allele-informative SNVs (see section “Materials and Methods”) were used and an in-house pipeline based on allele-informative SNVs was designed to identify ADE and MAE genes (Supplementary Figure 1 and section “Materials and Methods”). After filtering, 15,469 protein-coding genes (∼68.5% of protein-coding genes in the genome), in total, remained for downstream allelic gene expression analysis. The numbers of genes expressed in the eight tissues/cell types are similar, with an average number of 11,258, the highest number of 12,632 in lung, and the lowest number of 9,805 in liver (Supplementary Table 1). In contrast to this, the numbers of ADE genes we identified across tissues vary significantly. In cerebral cortex, we identified only 680 (5.48%) ADE genes which were less than half of the number observed in fibroblasts (13.92%, Supplementary Table 1). This is consistent with previous reports that brain is one of the most conserved organs between species (Zheng-Bradley et al., 2010). Similar to a previous study (Andergassen et al., 2017), we also observed bias toward higher expression of the C57BL/6J allele in ADE genes (Supplementary Table 1). To check whether the bias of allelic gene expression is caused by technical issues, we compared the log fold change (LFC) of allelic gene expression between tissues/cell types. If the allelic bias is largely caused by technical issues, we would observe similar correlations among different tissue pairs. As shown in Supplementary Figure 2A, the Spearman’s correlation coefficient between tissues ranges from 0.25 (between ESC and liver) to 0.55 (between lung and spleen). Such a big variation indicates that the number of ADE genes biased toward C57BL/6J may mainly represent a biological phenomenon rather than technical bias.

In each tissue, on average 27% of ADE genes show monoallelic expression (Figure 1C, Supplementary Table 1, and section “Materials and Methods”). Cerebral cortex contains fewer (∼18%) monoallelic genes than other tissues, while liver contains the highest percentage (∼33%) of monoallelic genes. Except for liver (394), the highest numbers of MAE genes were found in fibroblasts (409, 27.8%) and ESCs (362, 28.3%). These might partially represent clonally fixed random monoallelic expression, which cannot be easily detected in less homogenous tissues. Moreover, previous studies have shown that in hybrids the *M. spretus* X-chromosome is less likely to be inactivated than the *M. musculus domesticus* X-chromosome (Calaway et al., 2013). Our data are consistent with this prediction, with average *p* scores (see section “Materials and Methods”) for X-chromosomal genes ranging between 0.37 in heart and 0.49 in kidney.

### Monoallelic Genes Are Mostly Under Tissue-Dependent Regulation

**FIGURE 2:**
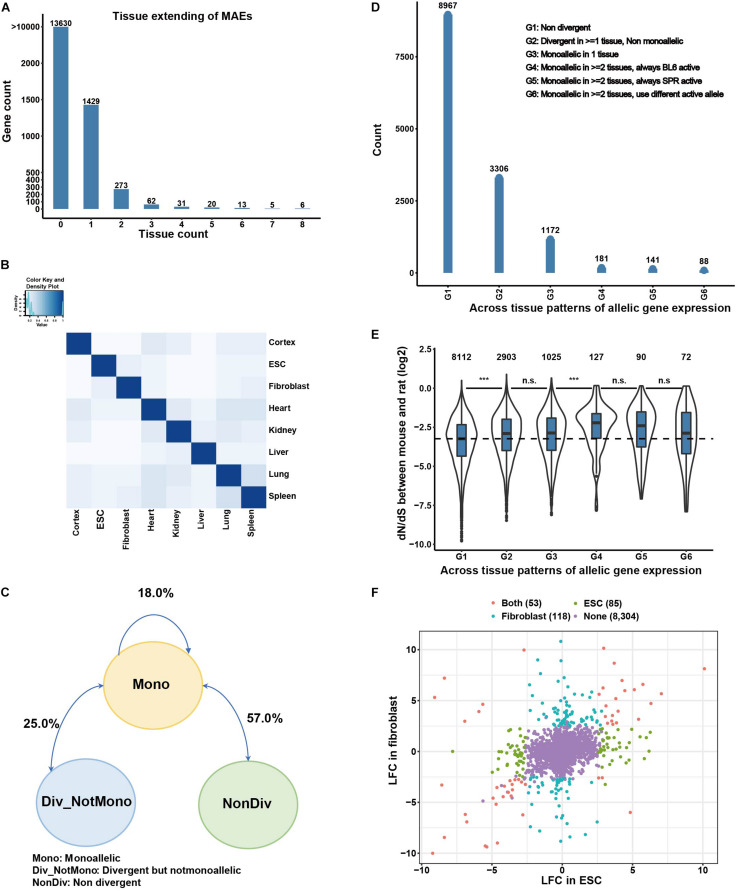
Monoallelic gene expression is mostly tissue-dependent. **(A)** Tissue distribution of monoallelic expressed genes. Zero in x axis means those genes are not MAEs in any of the analyzed tissues/cell types. **(B)** Replication rate of MAEs between tissues. Replication rates are measured by Jaccard Index (see section “Materials and Methods”). **(C)** Transition between different regulatory patterns. **(D)** Genes are classified into six groups based on their monoallelic patterns across tissues. G1 means the genes are non-divergent across tissues; G2 means the genes are divergent in more than one tissue but not monoallelic; G3 means the genes are monoallelic in only one tissue; G4 means the genes are monoallelic in more than one tissue and always BL6 allele active; G5 means the genes are monoallelic in more than one tissue and always SPR allele active; G6 means the genes are monoallelic in more than one tissue but with different active alleles. **(E)** dN/dS ratios of genes in different classes. **(F)** Comparison of monoallelic gene expression patterns between ESCs and fibroblasts.

*Cis*-regulatory divergence of monoallelic genes is also shaped by *trans*-factors in different tissues. To study this *cis*-*trans* interplay, we analyzed the tissue-dependent patterns of monoallelic gene expression (Figure 2A). Among the 1,839 MAE genes, most (1,429) are tissue specific, 404 genes are MAE in 2–7 tissues, and only 6 genes are monoallelic across all eight tissues. We also compared the replication rate of monoallelic genes between tissues by calculating a Jaccard index (see section “Materials and Methods”), for which also the direction of the expression bias is considered. As shown in Figure 2B, the replication rates for most tissue pairs are less than 20%, with a mean replication rate of 16.7%, and the maximal replication rate of 29.1% between spleen and lung. In comparison, the 125 known imprinted genes show higher consistency of allelic preference between tissues, as expected. Replication rates for these genes between tissues are mostly greater than 60%, and closely related tissues have higher replication rates; for example, the highest replication rate is 0.85 between lung and spleen (Supplementary Figure 2B). These results indicate that *cis*-regulatory monoallelic gene expression is pervasively tissue-dependent. Additionally, we found that the monoallelic status transitions mostly occur between monoallelic (”Mono” in Figure 2C) and non-divergent (”Non-Div” in Figure 2C), which comprised 57% of between tissue patterns, followed by the transition between divergent-but-not-monoallelic and monoallelic (∼25%, Figure 2C), and the remaining 18% between tissue patterns are the transitions between BL6-monoallelic and SPR-monoallelic. To further study the tissue-dependent patterns of monoallelic genes, we focused on genes expressed in two or more tissues/cell types. This group in total contains 13,855 genes, which we classified into six groups: (G1) genes are non-divergent in all expressing tissues; (G2) genes are divergent in at least one tissue but not monoallelic in any tissue; (G3) genes are monoallelic in only one expressing tissue; (G4) genes are monoallelic in two or more tissues and the BL6 allele is the active allele only; (G5) genes are monoallelic in two or more tissues and the SPR allele is the active allele only; (G6) genes are monoallelic in two or more tissues and with different active allele in different tissues. As shown in Figure 2D, except for the 8,967 (64.7%) non-divergent genes (G1), 3,306 genes (23.9%) belong to “G2,” 1,172 genes (8.5%) to “G3,” and 181 and 141 genes belong to “G4” and “G5,” respectively. In addition, we found 88 genes in “G6,” which have different active alleles in different tissues.

The *cis*-regulatory divergence of gene expression between species could be functional (adaptive evolution) or it could be noise caused by molecular error, as postulated before as “error hypothesis” (Zhang, 2018). Previous studies in alternative polyadenylation have found that most *cis*-regulatory divergence between species is noise caused by molecular error (Xu and Zhang, 2018). To test whether different tissue-dependent *cis*-regulatory monoallelic genes are under different selection constraint, we compared dN/dS ratios of genes among the six groups. As shown in Figure 2E, the dN/dS ratios of non-divergent genes are lower (G1 in Figure 2E) than those of divergent genes, no matter whether monoallelic or not. Among the genes with monoallelic patterns, those monoallelic in only one tissue have lower dN/dS ratios than those monoallelic in two or more tissues and with the same active allele between tissues. Interestingly, although statistically not significant, monoallelic genes with different active allele in different tissues have lower dN/dS ratios than those with the same active allele between tissues (Figure 2E), suggesting more complex regulatory patterns under this small set of genes.

To further explore the tissue-dependent *cis*-regulatory divergence and its underlying regulatory mechanisms, we put the focus on two cell lines (ESCs and fibroblasts), and performed Assay for Transposase-Accessible Chromatin with high-throughput sequencing (ATAC-seq) in samples of these two cell lines from our F1 hybrid mice (described in the next section). Comparing ESCs and fibroblasts (Figure 2F), there are 118 fibroblast-specific monoallelic genes, 85 ESC-specific monoallelic genes, and 53 genes monoallelic in both cell types. We confirmed with Sanger sequencing that allelic expression of the gene encoding apolipoprotein E (ApoE) is biased only in fibroblasts but not in ESC (Supplementary Figures 3A,B). And more interestingly, among the 53 genes monoallelic in both cell types, nine (∼17%) genes had different dominant alleles (with opposite direction of divergence) in the two cell lines. Again, by validating with Sanger sequencing (data not shown), two genes were confirmed, one is Msln with dominant BL6 allele in ESC and dominant SPR allele in fibroblasts. The other is Epb41l3 which, in contrast, has an active SPR allele in ESC and active BL6 allele in fibroblasts (Supplementary Figures 3A,B).

### Allelic Chromatin Accessibility Patterns in F1 ESCs and Fibroblasts

**FIGURE 3:**
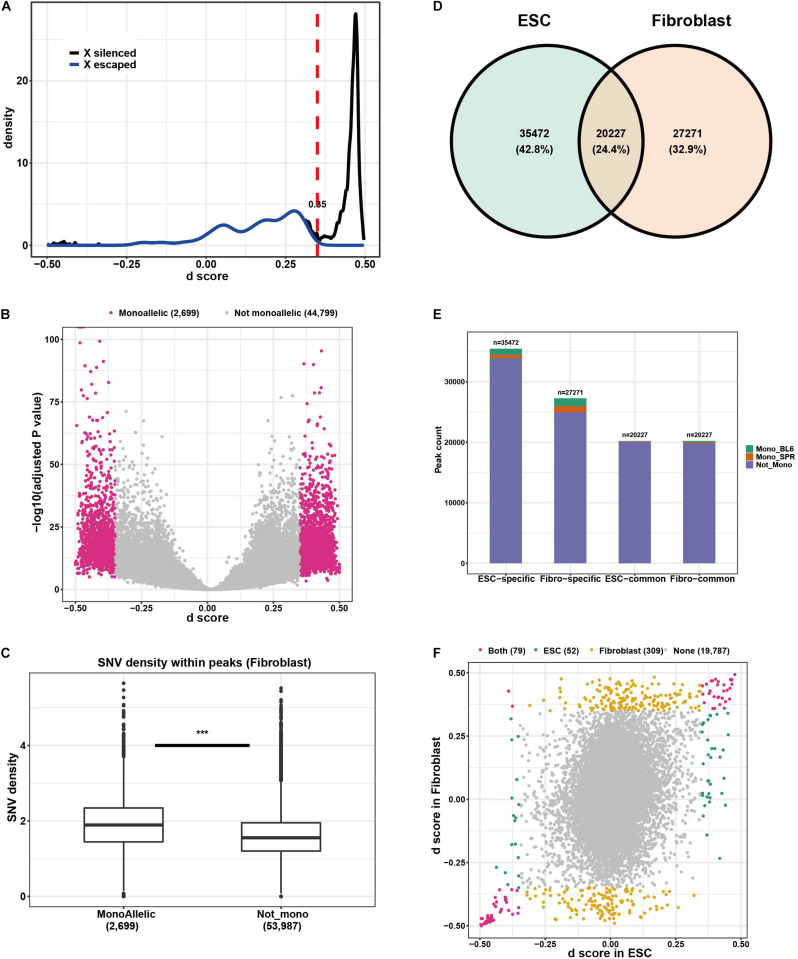
Quantification of allelic chromatin accessibility in ESCs and fibroblasts and cell-type dependent patterns. **(A)** *d* score of X-chromosome peaks in fibroblast. The blue line describes the distribution of *d* score of X-escaped peaks, black line indicates the *d* score distribution of X-silenced peaks. **(B)** Monoallelic peaks detected in fibroblast cells. **(C)** SNV density comparison between monoallelic peaks and non-monoallelic peaks. **(D)** Overlapped peaks between ESC and fibroblast. **(E)** Monoallelic peaks in cell type specific peaks and in peaks shared by the two cell types. **(F)** Cell type-dependent patterns of monoallelic peaks.

To understand tissue-dependent ADE patterns on the level of chromatin organization, we performed ATAC-seq experiments on six samples obtained from cultured ESC (three biological replicates) and fibroblasts (three biological replicates, see section “Materials and Methods”). Both replicates showed good correlation for ESCs and fibroblasts (Supplementary Figure 6). After processing the sequencing data (Supplementary Table 3) by following the pipeline described in Supplementary Figure 4, we identified 47,498 and 55,699 reproducible peaks in ESC and fibroblasts, respectively (Supplementary Table 4). When checking allelic read counts in each peak, fibroblast presents 4,247 (∼8.9%, Supplementary Table 3) allelic divergent peaks (ADP) which is nearly two times higher than ADPs in ESC (∼5.1%, Supplementary Table 3). To further identify monoallelic peaks, we calculated a *d* score (see section “Materials and Methods”) as defined before (Xu et al., 2017) for each peak, and use X-chromosomal peaks in fibroblast to determine the threshold of *d* score as 0.35 (Figure 3A and section “Materials and Methods”) for monoallelic peak identification. Based on this threshold, we identified 2,699 (∼5.7%, Figure 3B) monoallelic peaks in fibroblast, which is almost two times of the proportion in ESC (1,712 peaks, ∼3.1%, Supplementary Figure 5A). In addition to the differences in total number of monoallelic peaks between the two cell types, the ratio of components (C57BL/6J-active peaks and SPRET/EiJ-active peaks) is also different between ESC and fibroblasts. Among the 1,712 monoallelic peaks in ESC, 62.5% of them are C57BL/6J-active peaks and only 37.5% are SPRET/EiJ-active peaks, while in fibroblast, the two proportions are 55.9 and 44.1%, respectively (Supplementary Table 3). Since in the case of genetic-origin-dependent monoallelic expression the divergences between alleles are caused by *cis*-variants, we supposed that the SNV density in monoallelic peaks should be greater than that of non-monoallelic peaks. Indeed, as shown in Figure 3C, the median number of SNVs in monoallelic peaks in fibroblast cells is 1.89 per 100 base pairs, which is significantly higher than that in non-monoallelic peaks (with 1.56 median number of SNVs per 100 base pairs) in fibroblast. This is also observed in ESC (Supplementary Figure 5B). As shown in Figure 3D, most of the peaks are cell-type specific, only 24.4% of the identified peaks are shared between ESC and fibroblasts, while the others are either ESC-specific (42.8%) or fibroblast-specific (32.9%). And the monoallelic peaks are more likely to be found in cell-type specific peaks (Figure 3E). For those shared peaks, we also compared their divergence patterns between ESC and fibroblasts. Unlike the patterns of allelic gene expression (shown in Figure 2E), the bias of monoallelic peaks between tissues is much bigger (Figure 3F). Only 52 ESC-dependent monoallelic peaks were found, compared to 309 fibroblast-dependent monoallelic peaks. Among the 79 monoallelic peaks in both cell types, only 2 (∼2.5%) have different active alleles, which is much less than on the transcriptional level (17%).

### Integration of Allelic Gene Expression and Allelic Assay for Transposase-Accessible Chromatin-Peaks

**FIGURE 4:**
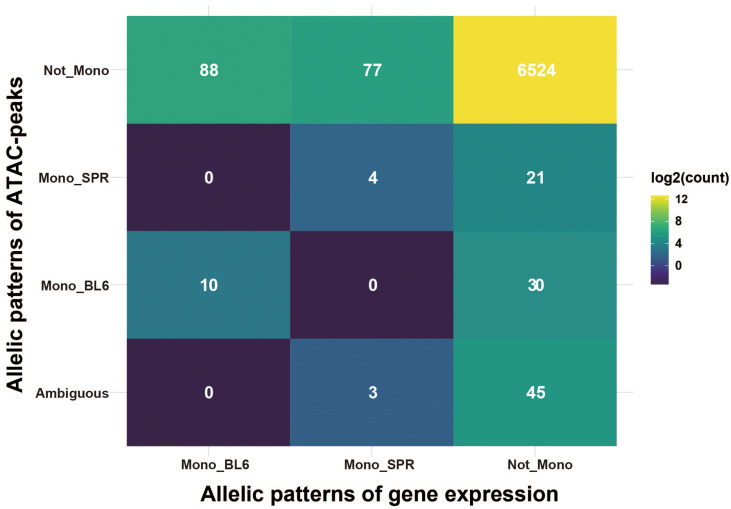
Integrated patterns of allelic gene expression and ATAC-peaks. The heatmap presents the integrated allelic patterns of gene expression and ATAC-peaks in its promoter regions (2.5 kb upstream and 0.5 kb downstream of TSS). Gene expression was classified into three classes: (1) Mono_BL6, means genes monoallelically expressed in C57BL/6J allele; (2) Mono_SPR, means genes monoallelically expressed in SPRETUS allele; (3) Not_Mono, means the genes are not monoallelic genes. ATAC-peaks are classified similarly with one more class indicated as “ambiguous” which means there are both “Mono_BL6” peaks and “Mono_SPR” peaks in that gene. Numbers in each cell indicate the count of overlapped genes. The color filled in cells are scaled by log2 of count.

To see the relationship of allelic patterns between transcription level (gene expression) and chromatin accessibility level (ATAC-peak), we integrated the two kinds of data by annotating peaks to promoter regions (2.5 kb upstream and 0.5 kb downstream of TSS) of target genes. Among the 10,559 genes expressed in fibroblasts, 6,802 (64.4%) contain at least one ATAC-peak in the promoter region. Interestingly, only 14 genes had consistent allelic patterns between gene expression and ATAC-peaks (Figure 4). In ESC, such cases are even fewer (three genes, Supplementary Figure 5C). This, on one hand, indicates that elements at promoter regions have limited contributions to allelic regulation of transcription, as reported before, distal elements like enhancers could play an important role. On the other hand, the allelic divergence at the transcription level may be invisible at the chromatin level. This is possible if the *cis* variants change the motif of one *trans* factor to another one without affecting chromatin organization. A previous study found that *cis*-regulatory mutations are more likely to change the binding motif of one transcription factor to that of another one than completely abolishing transcription factor binding (Payne et al., 2018), suggesting the plausibility of this mechanism.

## Discussion

In diploid eukaryotic organisms, the two alleles of each gene are generally expressed at similar levels. However, monoallelic gene expression occurs in various types and can be regulated by differential mechanisms involving genetic, epigenetic and/or stochastic elements. Classic monoallelic expression of X-chromosome-linked genes, olfactory receptor genes and developmentally imprinted genes has been documented elsewhere (Cedar and Bergman, 2008; Li and Sasaki, 2011; Schulz and Heard, 2013; Tucci et al., 2019). But the genetic-origin-dependent case including its regulatory mechanisms and especially evolutionary conservation among tissues is less studied until now. Here, we applied allelic RNA-seq and ATAC-seq to a highly polymorphic mouse hybrid F1 system crossed between the *M. musculus* strain C57BL/6J and the *M. spretus* strain SPRET/EiJ which possess the largest evolutionary distance to date in mouse to explore this case. Our study provides a detailed portrait of allelic gene expression including monoallelic genes, across multiple tissues/cell lines in a hybrid mouse model and allelic chromatin accessibility patterns in two different cell lines. We focus on depicting tissue-dependent allele-specific gene expression patterns and the underlying regulatory mechanisms at the chromatin accessibility layer. We observed pervasive tissue-dependent allele-specific gene expression and chromatin accessibility patterns. Cortex exhibited the fewest allele-specific expression differences while fibroblasts showed the most, which is consistent with previous results that the brain is one of the most conserved organs with regards to expression patterns (Zheng-Bradley et al., 2010). We identified six autosomal monoallelic genes with the active allele being identical in all eight tissues, resembling the patterns found for known imprinted genes and therefore likely to be novel candidates of imprinted genes. In addition, we found 88 monoallelic genes with different active alleles between tissues, which likely represent cases of genetic-origin-dependent MAE rather than random MAE. As shown in multiple previous studies, random monoallelic expression can be reliably detected in clonal cell lines (Eckersley-Maslin et al., 2014; Gendre et al., 2014). However, it is unlikely that the F1 hybrid tissues in our study are derived from a single clonal cell line. If this were the case, the X chromosome inactivation pattern would also show a biased pattern. Here, it is not the case, as shown in Supplementary Figure 7, the *p* scores (ranges from 0 to 1.0) calculated as the proportion of BL6 reads (*p* score close to 1.0 or close 0 indicates allele-specific expression, see details in section “Materials and Methods”) of the six F1 tissues are close to 0.5. In addition, we also observed high consistency between the two biological replicates for identified monoallelic genes (Supplementary Table 5), which further supports the mono allelic expression pattern was unlikely due to random inactivation of one allele. In contrast to the F1 tissues, the F1 ES cells and fibroblasts used in this study are clonal cell lines, and as expected we observe a *p* score in ES cells of about 0.5 (because X chromosome is not inactivated in ES cells) and a *p* score in fibroblasts of nearly 1.0. Therefore, some random monoallelic expression might be present in these two cell lines. However, as the number of monoallelic genes detected in these two cell lines is similar to that in the tissues (Figure 1C), we think even here the monoallelic expression is largely non-random. Our study is limited to identifying putative candidates for these two classes of MAE, as clonally fixed random MAE can only be detected in monoclonal cell lines, but not in any of the solid tissues, and single-cell experiments would be needed for detecting dynamic random MAE in the future. Interestingly, we found that for autosomal genes the C57BL/6J (maternal) allele is slightly more likely to be expressed at higher levels. Future studies should address the question whether this is a genome-wide genetic-origin-dependent (strain-specific) effect or a parent-of-origin effect comparable to that found by Crowley et al. (2015), who showed a global bias toward the paternal allele in *M. musculus* subspecies hybrids, and whether the preference of the maternal allele we found is unique to the interspecific cross used here.

We also elucidated the possible causal relationship between differential chromatin accessibility and gene expression. We observed that fibroblast cells had more monoallelic ATAC peaks than ESCs, suggesting ESCs are more conserved at the chromatin layer between these two strains. This finding agrees with the expectation that stronger selective constraints act on gene regulation in this early developmental stage than in fibroblasts. We also found that cell type dependent patterns similar to those at the gene expression level were also prominent at the chromatin layer. Additionally, C57BL/6J-active peaks were more prevalent than SPRET/EiJ-active peaks in both cell types, which corresponds to our data on the gene expression level, implying a potential parent-of-origin or strain-specific regulatory mechanism which needs future exploration. Finally, the *cis* regulatory mechanisms can partially account for the existence of monoallelic peaks, as the SNP density is higher in monoallelic peaks for both cell types. However, only a small part of monoallelic gene expression patterns could be explained by allelic chromatin structural patterns in promoter regions, suggesting that other distal elements may play important roles in shaping the patterns of allelic gene expression across tissues or that *cis*-regulatory mutations can change gene expression without affecting chromatin organization.

As reviewed in da Rocha and Gendrel (2019), DNA methylation, one major driver controlling gene expression, plays an important role in X chromosome inactivation and imprinting, but it is not a common epigenetic signature at loci with random monoallelic expression. Therefore, in the future, methods combining chromatin, DNA, RNA, protein and also single cell omics techniques will help to understand the interplay of hypermethylation and other molecular mechanisms for the regulation of different kinds of monoallelic expression, including genetic-origin-dependent monoallelic expression, and their relevance in speciation and phylogeny as well as health and disease.

## Data Availability Statement

The datasets presented in this study can be found in online repositories. The names of the repository/repositories and accession number(s) can be found below: Gene Expression Omnibus GSE176259, https://www.ncbi.nlm.nih.gov/geo/query/acc.cgi?acc=GSE176259.

## Author Contributions

BS, WL, and XZ developed the concept of the project and wrote the manuscript. WL performed the experiments. XZ analyzed the data with the help of BS, SZ, GL, and CT. All authors read and approved the final manuscript.

## Conflict of Interest

The authors declare that the research was conducted in the absence of any commercial or financial relationships that could be construed as a potential conflict of interest.

## Publisher’s Note

All claims expressed in this article are solely those of the authors and do not necessarily represent those of their affiliated organizations, or those of the publisher, the editors and the reviewers. Any product that may be evaluated in this article, or claim that may be made by its manufacturer, is not guaranteed or endorsed by the publisher.

## Footnotes

1
ftp://ftp.ensembl.org

2
http://www.sanger.ac.uk/

3
http://ensembl.org/

4
http://www.geneimprint.com/site/genes-by-species
