# Supplementary material for: Systematic Analysis of Monoallelic Gene Expression and Chromatin Accessibility Across Multiple Tissues in Hybrid Mice
Source: Front Cell Dev Biol. 2021 Sep 23;9:717555. doi: 10.3389/fcell.2021.717555 (PMC8495204; doi:10.3389/fcell.2021.717555)
Supplement: Supplementary file 1 [file Presentation_1.pdf]

FIGURE S1

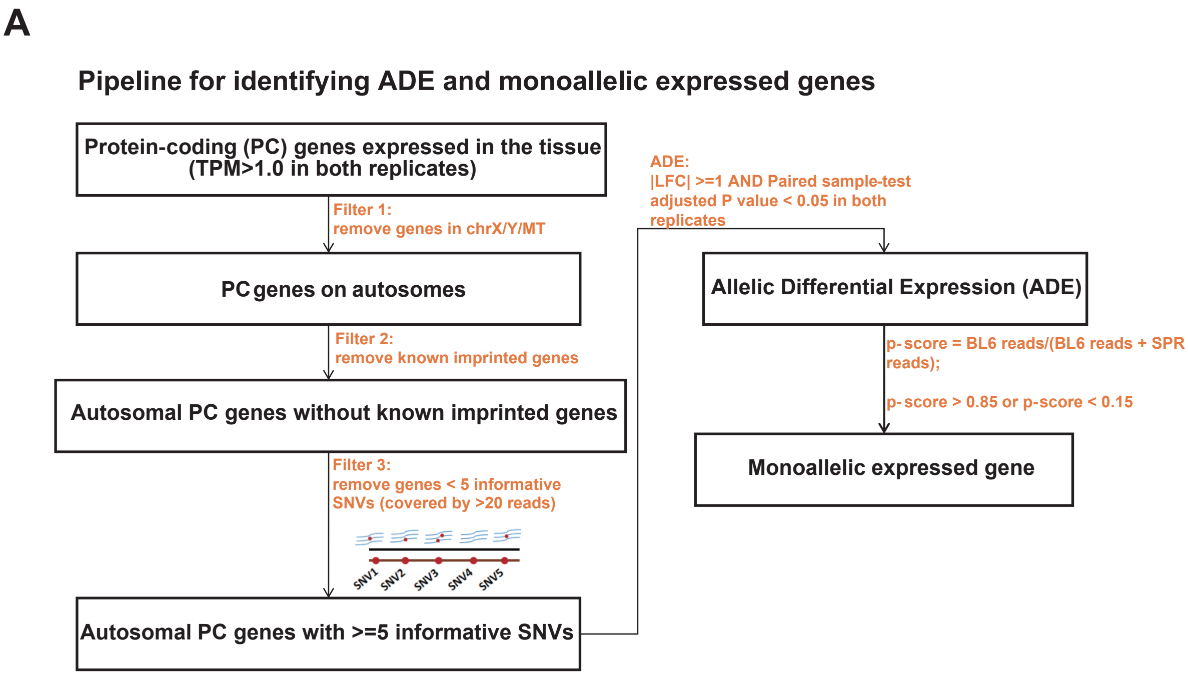
A
Pipeline for identifying ADE and monoallelic expressed genes
Protein-coding (PC) genes expressed in the tissue (TPM>1.0 in both replicates)
Filter 1:
remove genes in chrX/Y/MT
PC genes on autosomes
Filter 2:
remove known imprinted genes
Autosomal PC genes without known imprinted genes
Filter 3:
remove genes < 5 informative SNVs (covered by >20 reads)
SNV1
SNV2
SNV3
SNV4
SNV5
Autosomal PC genes with >=5 informative SNVs
ADE:
|LFC| >=1 AND Paired sample-test adjusted P value < 0.05 in both replicates
Allelic Differential Expression (ADE)
p-score = BL6 reads/(BL6 reads + SPR reads);
p-score > 0.85 or p-score < 0.15
Monoallelic expressed gene

FIGURE S2

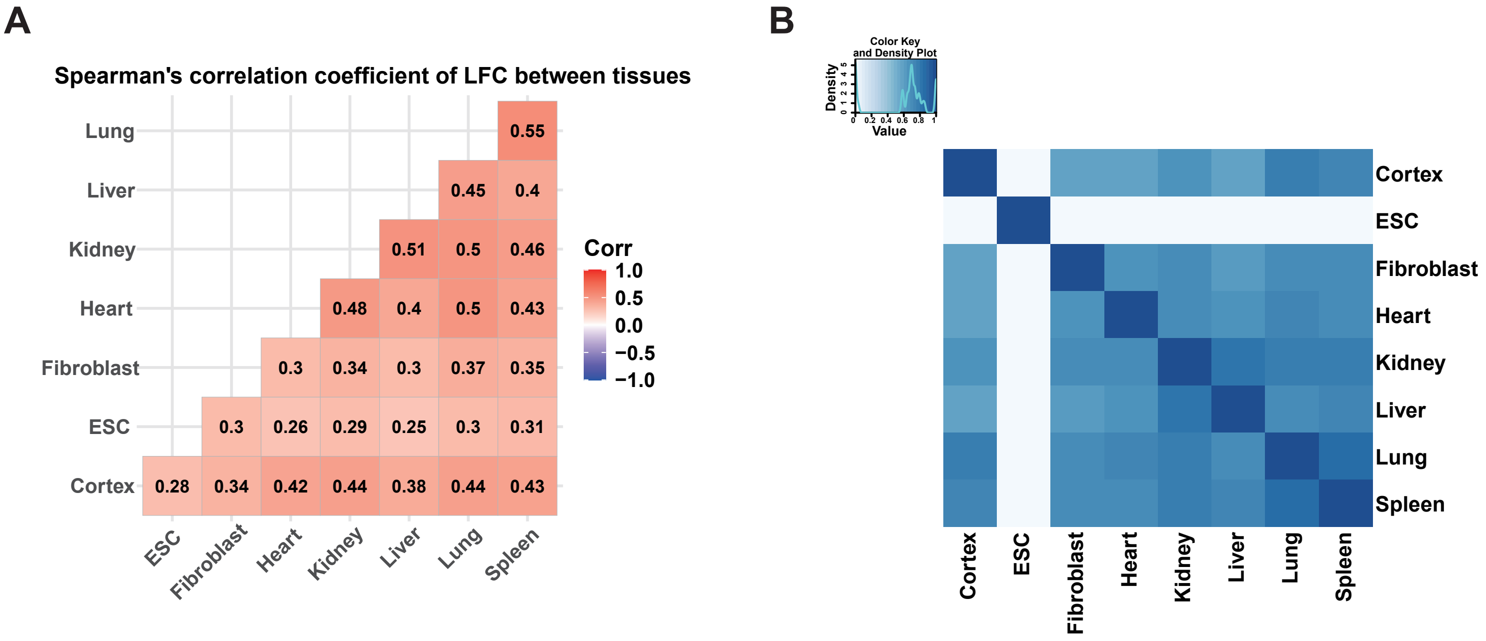
A
Spearman's correlation coefficient of LFC between tissues
Lung
Liver
Kidney
Heart
Fibroblast
ESC
Cortex
0.55
0.45 0.4
0.51 0.5 0.46
0.48 0.4 0.5 0.43
0.3 0.34 0.3 0.37 0.35
0.3 0.26 0.29 0.25 0.3 0.31
0.28 0.34 0.42 0.44 0.38 0.44 0.43
ESC Fibroblast Heart Kidney Liver Lung Spleen
Corr
1.0
0.5
0.0
−0.5
−1.0
B
Color Key
and Density Plot
Density
Value
Cortex
ESC
Fibroblast
Heart
Kidney
Liver
Lung
Spleen

FIGURE S3

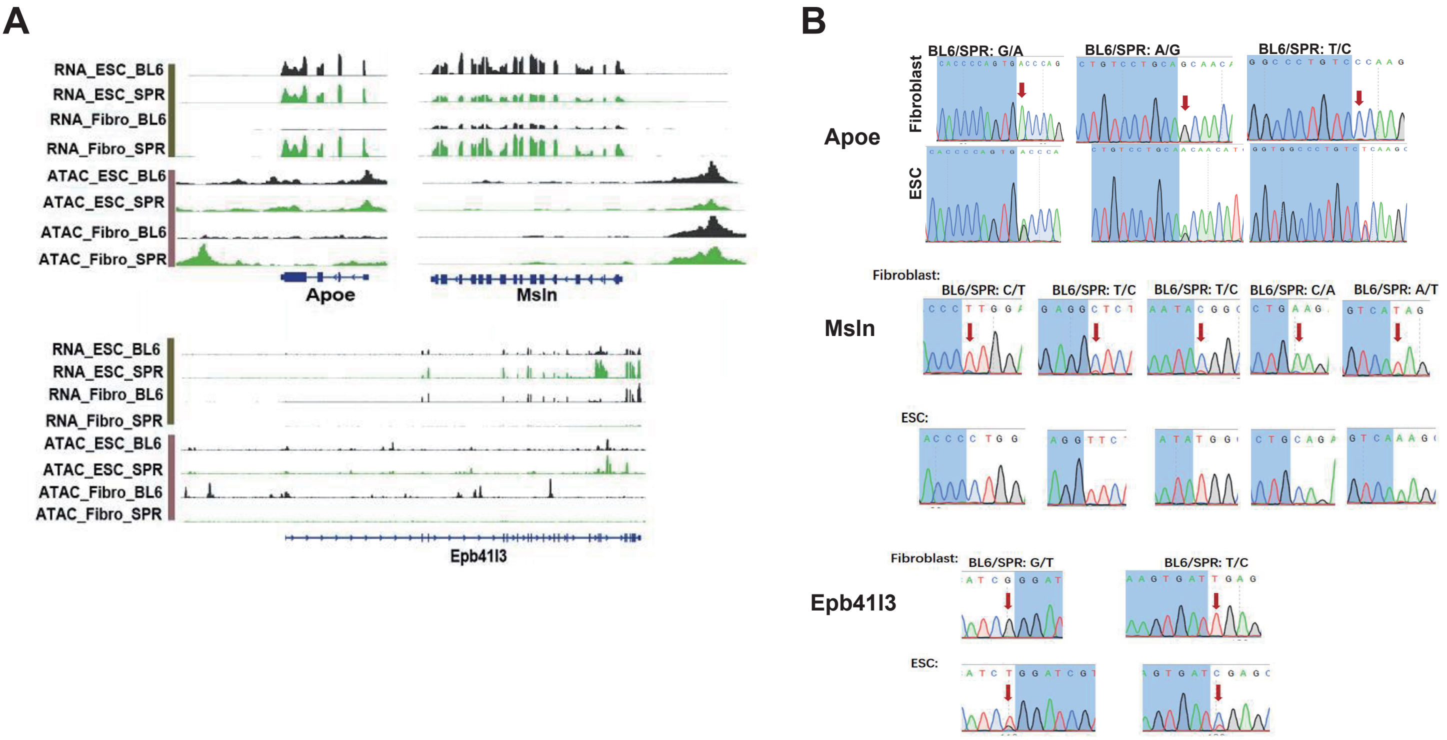
A
RNA_ESC_BL6
RNA_ESC_SPR
RNA_Fibro_BL6
RNA_Fibro_SPR
ATAC_ESC_BL6
ATAC_ESC_SPR
ATAC_Fibro_BL6
ATAC_Fibro_SPR
Apoe
Msln
Epb41l3
B
Apoe
Fibroblast
ESC
BL6/SPR: G/A
BL6/SPR: A/G
BL6/SPR: T/C
Msln
Fibroblast:
BL6/SPR: C/T
BL6/SPR: T/C
BL6/SPR: T/C
BL6/SPR: C/A
BL6/SPR: A/T
ESC:
Epb41l3
Fibroblast:
BL6/SPR: G/T
BL6/SPR: T/C
ESC:

FIGURE S4

A

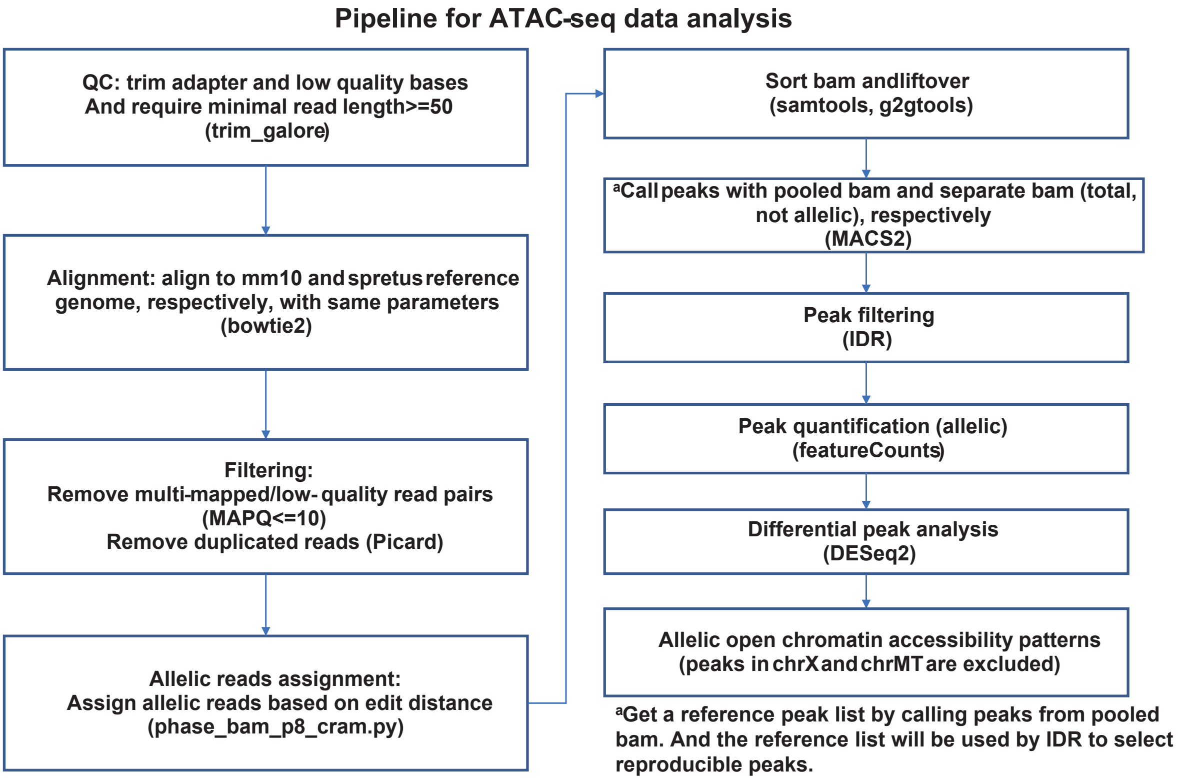
Pipeline for ATAC-seq data analysis
QC: trim adapter and low quality bases
And require minimal read length>=50
(trim_galore)
Alignment: align to mm10 and spretus reference genome, respectively, with same parameters
(bowtie2)
Filtering:
Remove multi-mapped/low- quality read pairs
(MAPQ<=10)
Remove duplicated reads (Picard)
Allelic reads assignment:
Assign allelic reads based on edit distance
(phase_bam_p8_cram.py)
Sort bam andliftover
(samtools, g2gtools)
aCall peaks with pooled bam and separate bam (total, not allelic), respectively
(MACS2)
Peak filtering
(IDR)
Peak quantification (allelic)
(featureCounts)
Differential peak analysis
(DESeq2)
Allelic open chromatin accessibility patterns
(peaks in chrX and chrMT are excluded)
aGet a reference peak list by calling peaks from pooled bam. And the reference list will be used by IDR to select reproducible peaks.

FIGURE S5

A

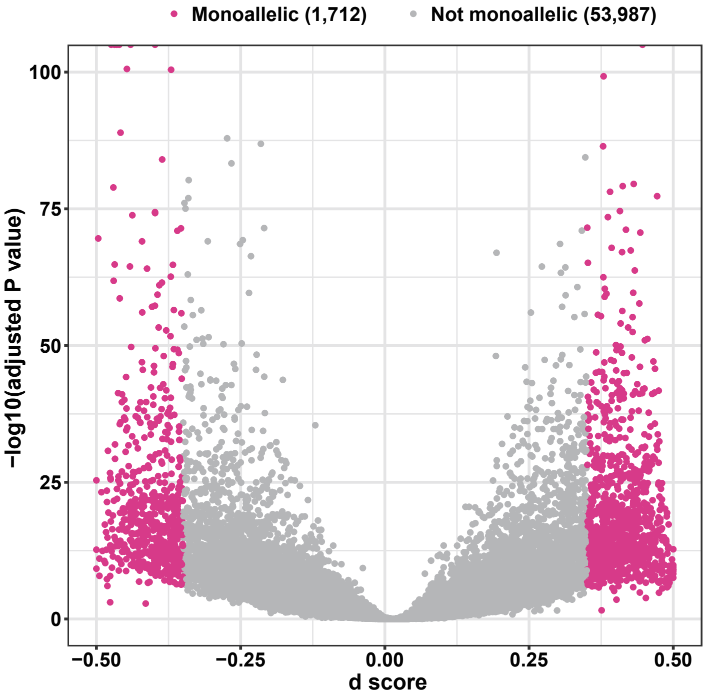

B

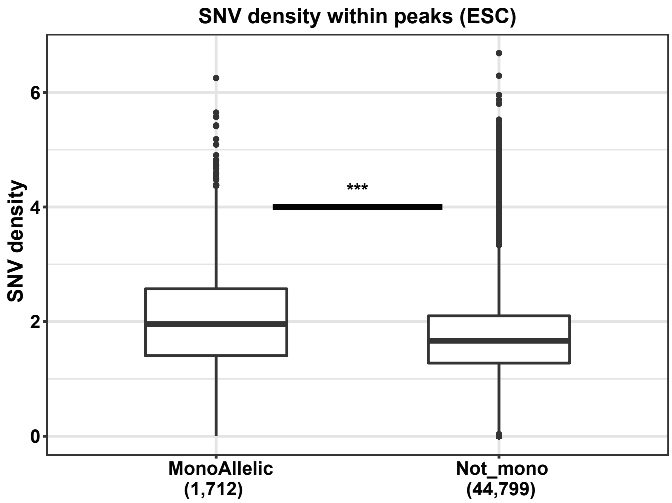

C

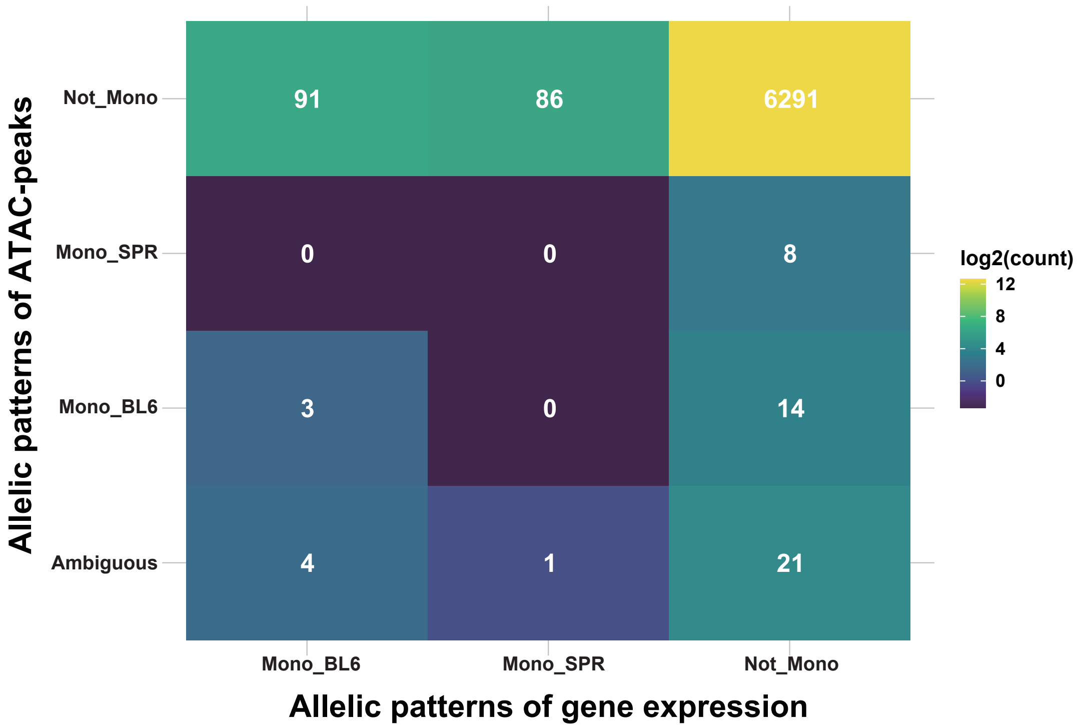

FIGURE S6

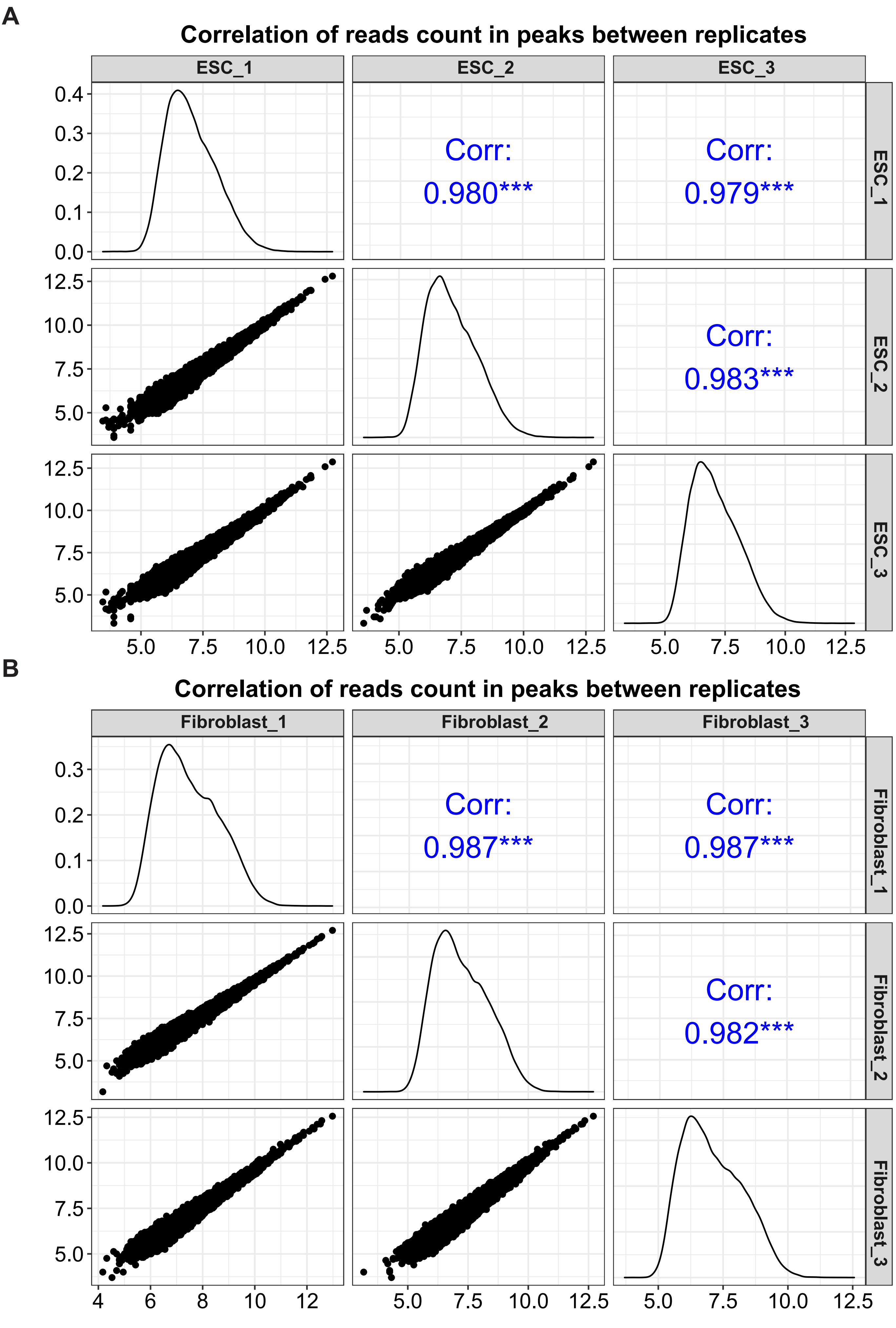
A
Correlation of reads count in peaks between replicates
ESC_1
ESC_2
ESC_3
Corr:
0.980***
Corr:
0.979***
Corr:
0.983***
0.0
0.1
0.2
0.3
0.4
5.0
7.5
10.0
12.5
B
Correlation of reads count in peaks between replicates
Fibroblast_1
Fibroblast_2
Fibroblast_3
Corr:
0.987***
Corr:
0.987***
Corr:
0.982***
4
6
8
10
12

FIGURE S7

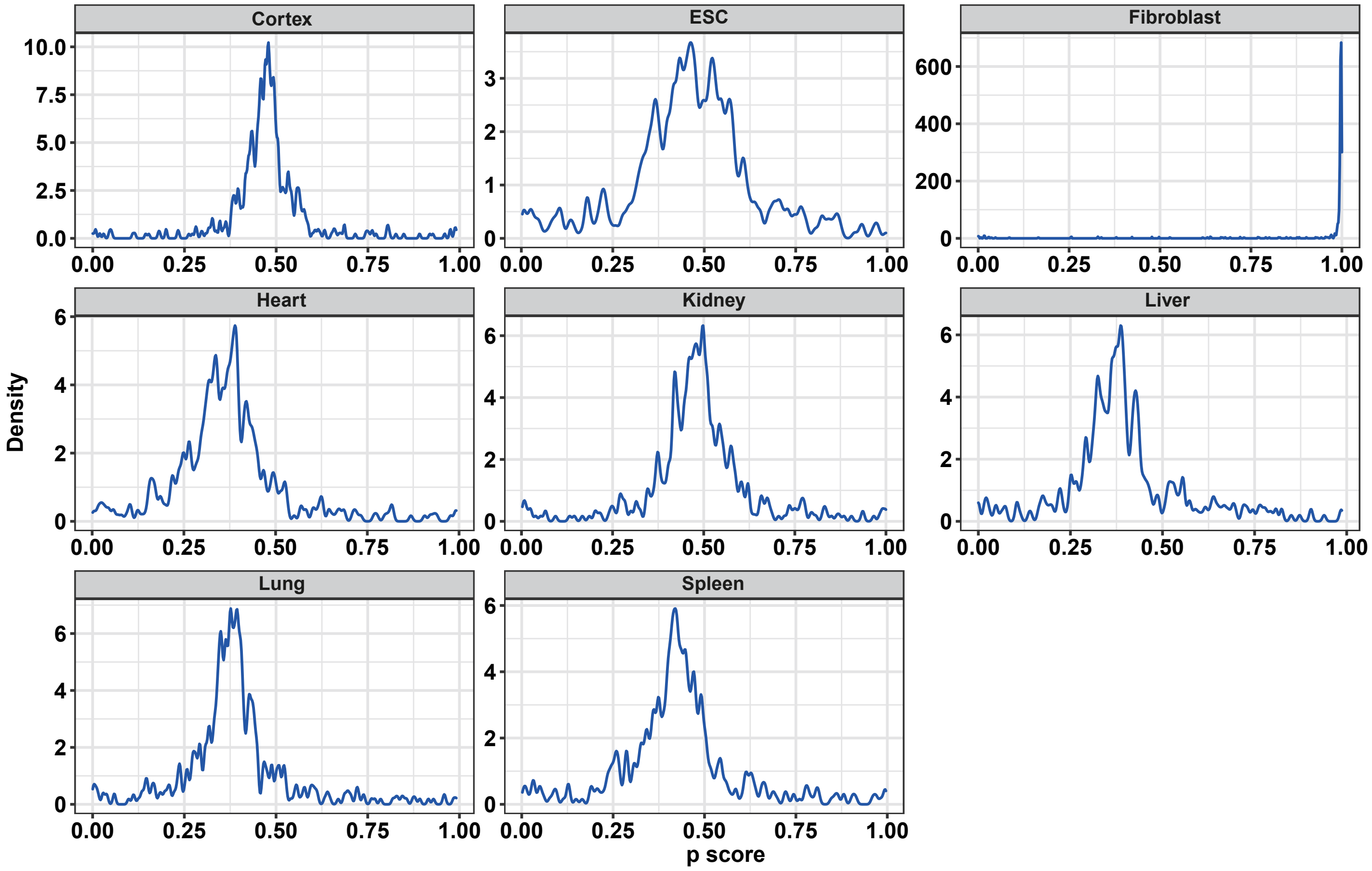

Cortex
ESC
Fibroblast
Heart
Kidney
Liver
Lung
Spleen
Density
p score
0.00
0.25
0.50
0.75
1.00
10.0
7.5
5.0
2.5
0.0
600
400
200

FIGURE S8

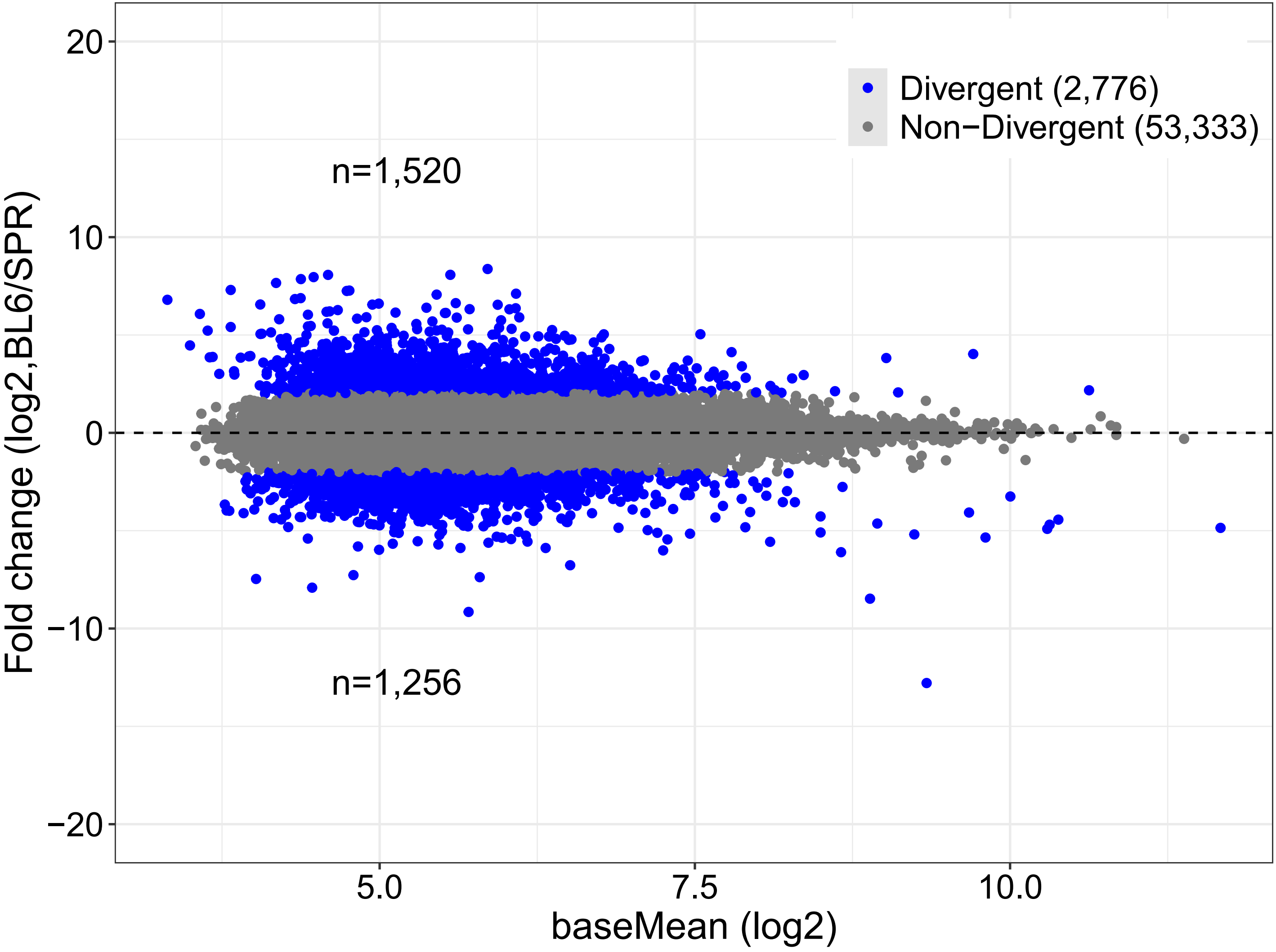
Divergent (2,776)
Non−Divergent (53,333)
n=1,520
n=1,256
20
10
0
−10
−20
Fold change (log2,BL6/SPR)
5.0
7.5
10.0
baseMean (log2)
