# Supplementary material for: Systematic Analysis of Monoallelic Gene Expression and Chromatin Accessibility Across Multiple Tissues in Hybrid Mice
Source: Front Cell Dev Biol. 2021 Sep 23;9:717555. doi: 10.3389/fcell.2021.717555 (PMC8495204; doi:10.3389/fcell.2021.717555)
Supplement: Supplementary file 2 [file Data_Sheet_1.docx]

Supplemental Figures S1-S5

Figure S1. (A)The pipeline for identifying allelic differentially expressed genes and monoallelically expressed genes.

Figure S2. Correlation of allelic divergence between tissues/cell lines and tissue replication of known imprinted genes. (A). Spearman’s correlation coefficient of LFC between tissues/cell lines. (B). Replication of known imprinted genes between tissues.

Figure S3. Three examples of tissue-dependent allelic expression and validation by Sanger sequencing. (A). IGV viewers of RNA-seq and ATAC-seq reads on Apoe, Msln, and Epb41l3. (B). Sanger sequencing experiments confirming the allelic patterns of the three genes (Apoe, Msln, and Epb41l3). Genotype of each informative SNV was labelled and the informative SNVs are marked by red arrows.

Figure S4. (A)The pipeline for ATAC-seq data analysis.

Figure S5. Allelic patterns of ATAC-peaks in ESC. (A). Monoallelic peaks detected in ESC. (B). SNV density comparison between monoallelic peaks and non-monoallelic peaks in ESC. (C). Integrated patterns of allelic gene expression and ATAC-peaks.

Figure S6. Correlation analysis of read counts in peaks between three replicates of ESCs and fibroblasts. (A) ESCs show good correlation between three replicates. (B) fibroblasts show good correlation between three replicates.

Figure S7. P-score analysis of X-chromosome genes in six tissues and two cell lines. The results suggested the p scores of the six F1 tissues are close to 0.5. In contrast to the F1 tissues, the F1 ES cells and fibroblasts used in this study are clonal cell lines, and as expected we observe a p score in ES cells of about 0.5 (because X chromosome is not inactivated in ES cells) and a p score in fibroblasts of nearly 1.0.

Figure S8. Down-sampling analysis on ES cells. The ATAC-seq reads of two alleles of ES cells were down-sampled to the same level to exclude alignment bias. The results show higher BL6 ATAC peaks also after equalizing read counts for both alleles.

Supplemental Tables

Table S1. Allelic gene expression quantification across tissues of F1 hybrid mice.

| F1 protein-coding | cortex | esc | fibroblast | heart | kidney | liver | lung | spleen | total |
| --- | --- | --- | --- | --- | --- | --- | --- | --- | --- |
| # exp | 14365 | 12908 | 12060 | 12356 | 13397 | 10994 | 14060 | 13217 | 17451 |
| autosomal genes with >5 informative^a^ SNVs in both replicates | 12775 | 11202 | 10925 | 11658 | 12450 | 10482 | 13182 | 12383 | - |
| With consistent allelic pattern between replicates | 12473 | 9895 | 10608 | 11171 | 11996 | 9844 | 12694 | 11802 | - |
| known imprinted genes removed | 12408 | 9860 | 10559 | 11114 | 11940 | 9805 | 12632 | 11747 | 15469 |
| Biallelic | 11728 | 8581 | 9089 | 10089 | 10705 | 8630 | 11349 | 10636 | - |
| ADE_BL6 | 387 (68) | 669 (186) | 831 (216) | 593 (151) | 646 (182) | 654 (227) | 773 (199) | 632 (169) | - |
| ADE_SPR | 293 (56) | 610 (176) | 639 (193) | 432 (117) | 589 (170) | 521 (167) | 510 (138) | 479 (131) | - |
| %ADE | 5.48% | 12.97% | 13.92% | 9.22% | 10.34% | 11.98% | 10.16% | 9.46% |  |
| %MAE (in ADE) | 18.23% | 28.30% | 27.82% | 26.15% | 28.50% | 33.53% | 26.27% | 27.00% |  |
| %MAE (in all expressed genes) | 1.0% | 3.67% | 3.87% | 2.41% | 2.95% | 4.02% | 2.67% | 2.55% |  |

^a^Informative SNV means the SNV is supported by more than 20 reads

^b^Consistent allelic pattern between replicates means the allelic divergence between the two replicates is in the same direction.

The numbers in parentheses represent the count of monoallelic expressed genes.

Table S2. Bias of allelic reads assignment.

| Sample | BL6_reads | SPR_reads | Common_reads | PCT_BL6 | PCT_SPR | PCT_Common | Delta_PCT (%) |
| --- | --- | --- | --- | --- | --- | --- | --- |
| Fibro_1 | 73841705 | 66138604 | 80067214 | 0.336 | 0.301 | 0.364 | 3.5 |
| Fibro_2 | 74889944 | 67039616 | 81573762 | 0.335 | 0.3 | 0.365 | 3.5 |
| Liver_1 | 78368641 | 74867101 | 73424058 | 0.346 | 0.33 | 0.324 | 1.6 |
| Liver_2 | 66965290 | 67525304 | 67783985 | 0.331 | 0.334 | 0.335 | -0.3 |
| Cortex_1 | 74358201 | 71212189 | 78751989 | 0.331 | 0.317 | 0.351 | 1.4 |
| Cortex_2 | 72637374 | 70690356 | 77290607 | 0.329 | 0.32 | 0.35 | 0.9 |
| Heart_1 | 68366479 | 66091077 | 89176790 | 0.306 | 0.296 | 0.399 | 1 |
| Heart_2 | 67083408 | 64964955 | 87154151 | 0.306 | 0.296 | 0.398 | 1 |
| Kidney_1 | 69608356 | 69387225 | 85460624 | 0.31 | 0.309 | 0.381 | 0.1 |
| Kidney_2 | 70994989 | 70650735 | 80761915 | 0.319 | 0.318 | 0.363 | 0.1 |
| ESC_1 | 48594298 | 47328575 | 55739396 | 0.32 | 0.312 | 0.368 | 0.8 |
| ESC_2 | 54509592 | 54649117 | 65118711 | 0.313 | 0.314 | 0.374 | -0.1 |
| Spleen_1 | 74549589 | 72141301 | 74381701 | 0.337 | 0.326 | 0.336 | 1.1 |
| Spleen_2 | 71425086 | 68908881 | 71562433 | 0.337 | 0.325 | 0.338 | 1.2 |
| Lung_1 | 88486235 | 84575419 | 78242006 | 0.352 | 0.337 | 0.311 | 1.5 |
| Lung_2 | 76014099 | 74001742 | 68589824 | 0.348 | 0.339 | 0.314 | 0.9 |

Table S3. ATAC-seq reads and mapping statistics.

| Sample | # Raw reads | # reads after trimming and filtering | % uniquely mapped to C57BL/6J | % uniquely mapped to SPRET/EiJ |
| --- | --- | --- | --- | --- |
| F1_Fibroblast_1 | 52565251 | 47061768 | 85.14% | 85.49% |
| F1_Fibroblast_2 | 48376374 | 42294318 | 84.30% | 84.62% |
| F1_Fibroblast_3 | 46862718 | 42373321 | 82.87% | 83.22% |
| F1_ESC_1 | 57697523 | 53218104 | 73.04% | 73.34% |
| F1_ESC_2 | 57862487 | 52794362 | 74.58% | 74.93% |
| F1_ESC_3 | 58137440 | 53133576 | 75.21% | 75.59% |

Table S4. ATAC-seq peaks identification and quantification.

|  | Fibro 1 | Fibro 2 | Fibro 3 | ESC 1 | ESC 2 | ESC 3 |
| --- | --- | --- | --- | --- | --- | --- |
| # Peaks in each sample | 107,209 | 105,221 | 87,208 | 169,716 | 160,355 | 145,100 |
| # reproducible peaks  (by IDR) | 48470 | | | 58056 | | |
| # peaks after filtering^a^ | 47498 | | | 55699 | | |
| #ADP_BL6 | 2211 (1510) | | | 1644 (1070) | | |
| #ADP_SPR | 2036 (1189) | | | 1204 (642) | | |
| % ADP | 8.9% | | | 5.1% | | |
| %Monoallelic in ADP | 63.6% | | | 60.1% | | |

^a^Filtering: peaks on X chromosome and mitochondria were removed, as were peaks on the end of chromosome 3 (>139M) and peaks on chromosome 14 .

The numbers in parentheses represent counts of monoallelic peaks.

Table S5. Proportion of consistently classified genes in each studied tissue or cell line.

| Tissue | Cortex | ESC | Fibroblast | Heart | Kidney | Liver | Lung | Spleen |
| --- | --- | --- | --- | --- | --- | --- | --- | --- |
| Proportion of consistently classified genes | 0.95 | 0.89 | 0.92 | 0.92 | 0.94 | 0.89 | 0.95 | 0.93 |
